# Significantly reducing blood loss via a peri-articular injection of tranexamic acid during total knee arthroplasty: a retrospective study

**DOI:** 10.1186/s12891-021-04591-0

**Published:** 2021-08-17

**Authors:** Yu-Kuan Lin, Shang-Wen Tsai, Po-Kuei Wu, Chao-Ming Chen, Jesse Chieh-Szu Yang, Cheng-Fong Chen, Wei-Ming Chen

**Affiliations:** 1 Department of Orthopaedics and Traumatology, Taipei Veterans General Hospital, Taoyuan branch, No. 100, Sec. 3, Cheng-Kung Road, 330 Taoyuan, Taiwan, Republic of China; 2 Department of Orthopaedics and Traumatology, Taipei Veterans General Hospital, 18F, No. 201, Sec. 2, Shih-Pai Road, 112 Taipei, Taiwan, Republic of China; 3 College of Biomedical Science and Engineering, National Yang Ming Chiao Tung University, No. 155, Sec. 2, Linong Street, 112 Taipei, Taiwan, Republic of China; 4 Orthopaedic Department School of Medicine, National Yang Ming Chiao Tung University, No. 155, Sec. 2, Linong Street, 112 Taipei, Taiwan, Republic of China; 5 Institute of Clinical Medicine, School of Medicine, National Yang Ming Chiao Tung University, No. 155, Sec. 2, Linong Street, 112 Taipei, Taiwan, Republic of China

**Keywords:** Peri-articular injection, Tranexamic acid, Total knee arthroplasty, Blood loss, Complication

## Abstract

**Background:**

The administration of an intra-articular injection (IAI) of tranexamic acid (TXA) has been demonstrated to be effective in reducing both blood loss and transfusion rate during total knee arthroplasty (TKA); however, few studies have reported the efficiency of a peri-articular injection (PAI) of TXA. We studied the efficiency of a PAI of TXA in reducing blood loss during TKA.

**Methods:**

Fifty patients undergoing primary simultaneous bilateral TKA were enrolled in this retrospective study. The right knee received a PAI of 1 g of TXA (Group I), and the left knee received an IAI of 1 g of TXA (Group II). The clinical outcome measures were a change in blood loss from Hemovac drains and surgical time.

**Results:**

The decrease in blood loss from the Hemovac was significantly lower in Group I (460.1 ± 36.79 vs. 576.0 ± 34.01, *P* < 0.001) than in Group II, and no significant difference in surgical times was observed. The blood transfusion rate in the present study was 16 %.

**Conclusions:**

A PAI of TXA may reduce blood loss more efficiently than an IAI of TXA during TKA without increased complications such as surgical site infection, poor wound healing, skin necrosis, pulmonary embolism, and deep vein thrombosis.

## Background

Knee osteoarthritis is an increasingly common disease that causes knee pain, a limited range of motion, reduced quality of life, and a reduced ability to perform activities of daily living. Total knee arthroplasty (TKA) is a reliable surgical method for treating these symptoms; however, postoperative blood loss is a major risk factor for TKA. Studies conducted between 2000 and 2009 have reported blood loss of 1000–1790 mL per patient during unilateral TKA and an increase in the incidence of allogeneic blood transfusions [1–4]. Increased blood loss and a higher incidence of complications such as anemia, weakness with delayed rehabilitation, and poor wound healing have previously been noted [5]. Although allogeneic blood transfusions are a safe and effective treatment for considerable postoperative bleeding, blood transfusions pose potential risks, including immunological allergy, infection, and mortality, as well as lengthy hospital stays and increased financial costs. Therefore, numerous approaches have been developed to reduce the amount of blood loss and the rate of blood transfusion, such as minimally invasive surgery, the use of a tourniquet during surgery, and hypotensive anesthesia. In addition, the use of intravenous and intra-articular tranexamic acid (TXA) has been reported to safely and effectively reduce blood loss and the likelihood of blood transfusions in TKA [6–8].

Aguilera et al. [9] reported that the topical administration of 1 g of TXA reduced blood loss in TKA, and Yozawa et al. [10] reported that a peri-articular injection of 1 g TXA effectively reduced blood loss compared with placebo treatment in TKA. Aside from these two reports, there are few studies on the efficacy of a peri-articular injection (PAI) of a local pain control cocktail combined with TXA in TKA [10–12]. Therefore, in this study, we compared blood loss during simultaneous bilateral TKA when one knee received a peri-articular TXA injection and the other knee received an intra-articular TXA injection. We hypothesized the PAI of TXA may reduce blood loss more efficiently than an IAI of TXA.

## Methods

A total of 50 patients who underwent simultaneous bilateral TKA for advanced knee osteoarthritis between January and August 2017 were included in this retrospective study, which was approved by the Institutional Review Board of our hospital, Taipei Veterans General Hospital (IRB number: 2018-09-004CC). The inclusion criteria were age ≥ 60 years, a formal diagnosis of advanced knee osteoarthritis and received bilateral TKA. The exclusion criteria were a history of knee surgery, inflammatory arthritis, osteoarthritis due to infection, unilateral TKA, renal failure, and bleeding or platelet disorders. Patient demographic data such as age, sex, body mass index (BMI), and the Charlson Comorbidity Index were also included [13].

In this simultaneous bilateral TKA study, Group I comprised the right knees that received a PAI of TXA (1 g/10 mL), and Group II comprised the left knees that received an IAI of TXA (1 g/10 mL).

The surgical procedures were performed by a single experienced joint surgeon. During surgery, the patients were placed in a supine position under spinal anesthesia. Before skin incision, a tourniquet was inflated until the pressure reached 280 mmHg. A curved skin incision was made using the midvastus approach. The prosthesis used was a cement-fixed NexGen posterior-stabilized knee system (Zimmer Biomet). The sites of the PAI were the rectus femoris, vastus medialis, patella tendon, pes anserinus, and posterior capsule. Surgical drains were inserted into the joints and clamped, opened 1 h after surgery, suctioned using full-negative pressure, and removed 48 h after surgery. The tourniquet was then deflated after the PAI or IAI of TXA. Standard wound closure was performed, and a sterile dressing was applied. The procedures were performed using a PAI of TXA on the right knee first and then an IAI of TXA on the left knee.

The postoperative venous thromboembolism prophylaxis didn’t use in patients, except for those with a BMI greater than 30 kg/m^2^. These patients received an injection of low-molecular-weight heparin at 2000 international units (IU) daily for 3 days to prevent thromboembolism. Hemoglobin (Hb) levels of all patients were rechecked on the first postoperative day. The criteria for blood transfusion were Hb < 8 mg/dL and a decrease in the Hb level of > 3.0 mg/dL if the patient had intolerable symptoms or organ dysfunction due to anemia. All patients underwent mobilization on the first postoperative day and were discharged 5 days after surgery.

Clinical outcomes included blood loss from Hemovac drains, surgical time, and transfusion rate.

All analyses were performed using IBM SPSS Statistics 22.0 (IBM, Armonk, NY, USA). The characteristics of the two groups were analyzed using two sample t test (surgery time, intraoperative blood loss, and blood loss from Hemovac drains). Statistical significance was set at p < 0.05.

## Results

Between January and August 2017, 50 patients who met the inclusion criteria were enrolled for simultaneous bilateral TKA. The right knees (Group I) received a PAI of TXA (1 g/10 mL), whereas the left knees (Group II) received an IAI of TXA (1 g/10 mL).

Due to simultaneous bilateral TKA, we could not compare basic patient characteristics and blood examination data, including age, sex, BMI, Charlson index score, pre- and postoperative Hb, Hb change, and transfusion rate, between the PAI and IAI groups (Table 1). The comparisons of surgical time and Hemovac drain volume between the two groups are shown in Table 2.

**Table 1:** Patient demographic and pre- and postoperative data

Number	50
Age (years)	70.46 ± 1.26
Sex	
Male/Female	36.0 % (18/50) / 64.0 % (32/50)
BMI (kg/cm^2^)	27.65 ± 0.46
ASA score (number/%)	
1	0
2	28 (56 %)
3	22 (44 %)
4	0
5	0
Charlson index score	1.30 ± 0.15
Hb preoperative (mg/dL)	12.77 ± 0.24
PLT count preoperative(count x 10^3^/mm^3^)	217.6 ± 7.89
Hb postoperative (mg/dL)	10.51 ± 0.23
Hb change (mg/dL)	-2.26 ± 0.21
Transfusion rate (number/%)	8 (16 %)

**Table 2:** Comparison of data between PAI and IAI

	Peri-articular injection (Group I)	Intra-articular injection (Group II)	*P* value
Number	50	50	
Surgery time	33.96 ± 0.73	34.9 ± 0.83	0.065
Intraoperative blood loss (mL)	34.66 ± 1.41	39.42 ± 0.97	0.472
Hemovac volume (mL)	460.1 ± 36.79	576.0 ± 34.01	< 0.001

Comparing Group I and Group II, the Hemovac volume was significantly lower in Group I (460.1 ± 36.79 mL vs. 576.0 ± 34.01 mL, *P* < 0.001). The mean surgical times and intraoperative blood loss of the two groups did not significantly differ (33.96 ± 0.73 vs. 34.9 ± 0.83 min, and 34.66 ± 1.41 vs. 39.42 ± 0.97 mL).

The Hb level change of the patients was − 2.26 ± 0.21 mg/dL, and the transfusion rate was 16 % (8/50). None of the patients had major or minor complications such as deep vein thrombosis or pulmonary embolism, surgical site infection, poor wound healing, and skin necrosis.

## Discussions

In our study, a PAI with 1 g TXA was more efficient at reducing blood loss in simultaneous bilateral TKA than an IAI with 1 g TXA. According to reports, an IAI of TXA results in significant reductions in total blood loss, blood loss via the surgical drain, and Hb decrease [2, 8, 14–16]. Seo et al. reported that the transfusion frequencies after placebo, intravenous injection, and an IAI of TXA were 94 %, 34 %, and 20 %, respectively, and that these rates significantly differed between the intravenous injection and IAI in the TXA groups [14].

The blood loss noted after TKA presented as bone oozing and soft-tissue oozing. The studies on the IMI or PAI of TXA in unilateral TKA are limited [10–12]. Mao et al. demonstrated that an IMI and IAI of TXA efficiently reduced blood loss during TKA compared with a control group; however, the difference between the IMI and IAI was nonsignificant [11]. Pinsornsak et al. reported that the antibleeding effects of intramuscularly injected and intravenously infused TXA were similar [12]. Yozawa et al. reported that compared with placebo treatment, a PAI of TXA was efficient at decreasing blood loss and decreasing Hb and hematocrit [10].

In our study, the PAI of TXA was more efficient at reducing blood loss in simultaneous bilateral TKA than the IAI of TXA. Comparing Groups I and II, the Hemovac drain volumes of the PAI group (Group I) were significantly lower than those of the IAI group (Group II) (460.1 ± 36.79 mL vs. 576.0 ± 34.01 mL, *P* < 0.001). The blood transfusion rate was 16 % on simultaneous bilateral TKA in our study, which is lower than that in the study by Seo et al. on unilateral TKA (20 %) [14].

Our study had five limitations. First, the coagulation ability may influence the amount of blood loss during the procedure. All patients in our study underwent right TKA first, followed by left TKA. Second, the optimal dose of TXA was not clarified. Third, due to simultaneous bilateral TKA in our study, we could not compare the total blood loss and blood transfusion rate. Fourth, we could not clarify the influence of the negative pressure drain between the PAI and IAI groups. Fifth, we used the different threshold of blood transfusion.

## Conclusions

The PAI of TXA may efficiently reduce blood loss compared to the IAI of TXA during TKA without causing deep vein thrombosis, pulmonary embolism, or skin problems. More studies to clarify the limitations in our study are needed.

## Data Availability

The datasets analyzed during the current study are not publicly available because the data and materials were accessed from the case system of our department. They are available from the corresponding author upon request.

## Abbreviations

PAI: Peri-articular injection
TXA: Tranexamic acid
TKA: Total knee arthroplasty
IAI: Intra-articular injection
BMI: Body mass index
ASA: American Society of Anesthesiologists
Hb: Hemoglobin
Plt: Platelet
